# Dietary Contributors to Food Group Intake in Preschool Children Attending Family Childcare Homes: Differences between Latino and Non-Latino Providers

**DOI:** 10.3390/nu12123686

**Published:** 2020-11-29

**Authors:** Andrea Ramirez, Maya Vadiveloo, Patricia M. Risica, Kim M. Gans, Mary L. Greaney, Noereem Z. Mena, Kristen Cooksey Stowers, Alison Tovar

**Affiliations:** 1 Department of Nutrition and Food Sciences, University of Rhode Island, Kingston, RI 02881, USA; maya_vadiveloo@uri.edu (M.V.); mnoereem@gmail.com (N.Z.M.); alison_tovar@uri.edu (A.T.); 2 Department of Behavioral and Social Sciences, Brown University School of Public Health, Providence, RI 02912, USA; patricia_risica@brown.edu; 3 Department of Human Development & Family Sciences, University of Connecticut, Storrs, CT 06269, USA; kim.gans@uconn.edu; 4 Department of Health Studies, University of Rhode Island, Kingston, RI 02881, USA; mgreaney@uri.edu; 5 Department of Allied Health Sciences, University of Connecticut, Storrs, CT 06269, USA; kristen.cooksey@uconn.edu

**Keywords:** childcare, family childcare homes, providers, ethnicity, diet quality, food groups

## Abstract

While there are several factors that contribute to the diet quality of children in childcare, one contributing factor in Family Childcare Homes (FCCHs) is the provider’s ethnicity. However, research examining the food items provided in this setting is limited; in particular, with regards to differences between FCCHs of Latino and non-Latino providers. The aim of this study was to identify and describe the food items that contribute to food group intake in preschool-aged children attending FCCHs, and to examine differences by provider ethnicity. This secondary data analysis used baseline data from Healthy Start/Comienzos Sanos: a cluster-randomized trial. Children’s dietary intake was collected using the Dietary Observation in Child Care method and entered into Nutrition Data System for Research software. Food groups were based on the Nutrition Coordinating Center classification. Contribution of food items to their respective food group was calculated as a proportion, using ratio of means and presented as a percentage. Ethnic differences were tested with ANCOVA (*p* < 0.05) with Bonferroni adjustments for multiple comparisons. All providers (*n* = 120) were female and 67.5% were Latino. Most fruit consumed by children was in the form of juice (85%), three-fourths of the grains consumed were refined (75%), and half of the sweets consumed were syrup/honey/jelly (50%). Most of the vegetables consumed were non-starchy (61%), nearly three-fourths of dairy consumed was low-fat (71%), and vegetable oils contributed the most to the fats group (89%). Food items differed by provider’s ethnicity, with children cared for by non-Latino providers consuming a higher proportion of fruit juice, animal fats and a lower proportion of legumes (*p* < 0.001 for all). Children with Latino providers consumed a lower proportion of non-starchy vegetables, low-fat dairy, and nuts/seeds (*p* < 0.001 for all). FCCH providers could offer more whole fruits and grains and a greater variety of vegetables. Differences by ethnicity suggest providers could benefit from culturally tailored recommendations.

## 1. Introduction

The preschool years are a critical period for the development of food preferences and food eating patterns [1,2,3]. Exposing children to a variety of nutrient-dense foods and limiting less nutritious ones is essential for the development and reinforcement of healthy eating patterns [4]. Eating patterns formed during these early years can extend across the lifespan [1,2,3]. Thus, ensuring healthy environments where children spend most of their time represents an opportunity to improve health and prevent chronic diseases later in life [5,6,7,8].

In the United States (US), three-quarters of preschool-aged children are enrolled in some type of childcare [9], where they spend on average 33 h per week [10]. This includes Family Childcare Homes (FCCHs), a specific type of childcare where non-relative providers care for the children in their own home instead of a separate facility [11]. Nearly 2 million children in the US are enrolled in FCCHs [10], which have become a primary resource for low-income and ethnically diverse families [12]. 

Given the increased reliance of parents on FCCHs, these are an important environmental and social setting that can influence the development of dietary habits [10,13,14,15]. In the FCCHs, providers are responsible for offering healthy food to children in their care [16]. Therefore, providers can become an influential figure in shaping children’s food preferences by exposing them to a wide variety of fruits and vegetables, high quality, nutrient-dense foods, and limit consumption of low-nutrient, energy-dense foods and beverages [16,17]. Several factors influence the providers’ food selection including participation in the Child and Adult Care Food Program (CACFP) [18,19], which is a federal reimbursement program for meals and snacks to eligible childcare centers and FCCHs [13,19,20]. In order to get reimbursed, childcare providers must follow the CACFP meal pattern guidelines, which are consistent with the Dietary Guidelines for Americans (DGA) [21]. 

In addition to participating in the CACFP, FCCH providers (FCCPs) are ethnically diverse [22], and their ethnicity has been related to different dietary practices in childcare settings [23,24]. Research also suggests that cultural and social differences play a role in the foods offered to children [22,25,26]. One study reported that for Latino FCCPs, culture influenced the foods they served in childcare; however, differences between food items were not explored in depth [17]. In the US, diet quality and eating patterns have differed by ethnicity. Two studies using data from the National Health and Nutrition Examination Survey (NHANES) found that Hispanics had higher total diet quality scores compared to whites and non-Hispanic Blacks, for both adults and children [27,28]. Furthermore, other studies found that total diet quality scores were highest among Mexican Americans and lowest among non-Hispanic Black participants [29]. These reported differences in diet quality were not specific to childcare; however, these ethnic variations could be translated to these settings. A previous analysis of data from the Healthy Start study, found that children under the care of Latino FCCPs had higher diet quality (Healthy Eating Index: 64.4) compared to non-Latino FCCPs (Healthy Eating Index: 56.6). While this study found that children under the care of Latino FCCPs had higher component diet quality scores for greens/beans, total protein, and seafood and plant protein, this analysis did not take into account the specific types of foods offered and the ethnic differences in the foods provided [24]. While the CACFP is a very important predictor of the nutritional quality of foods served in childcare [30,31,32,33], exploring other factors among FCCHs that care for underserved children is needed. This is particularly important given that children still do not meet recommendations for fruits, vegetables, whole grains and low-fat milk, and are consuming too many sugar-sweetened beverages (SSBs), fruit juice and snacks, with excess sugars, saturated fat and sodium [34,35,36,37,38,39,40,41]. Furthermore, many of these studies have been conducted in childcare centers, and even though FCCHs provide care of a substantial number of preschoolers, studies exploring what children are consuming in FCCHs are limited [42]. While some studies have been conducted in FCCHs, these have assessed overall diet quality and major food groups [12,22,42,43,44,45], and have not examined the specific food items that contribute to major food groups and have not looked at differences in specific food sources by FCCPs ethnicity. 

This information is necessary to develop more effective, culturally targeted interventions, training, and education [17,22]; however, research examining the differences in foods between Latino and non-Latino FCCPs is limited. Therefore, the aim of this study was to identify and describe the food items that contribute to food group intake in preschool age children attending FCCHs, and to examine differences by FCCPs ethnicity. 

## 2. Materials and Methods

This study was a cross-sectional, secondary analysis of baseline data from Healthy Start/Comienzos Sanos, a cluster-randomized controlled trial of a multicomponent intervention designed to promote healthy nutrition and physical activity behaviors of children attending English- and Spanish-speaking FCCHs. FCCHs were randomized in matched pairs, by FCCPs language spoken and by the number of eligible children, into the intervention (nutrition and physical activity) or the comparison group (reading readiness) [46]. The study included 120 FCCPs and 374 enrolled children between 2 and 5 years of age.

FCCPs were eligible for the study if their FCCH was within 60 miles of Providence, Rhode Island; had been in operation for at least 6 months with at least one child taken care of for a minimum of 10 h per week; served one meal and snack [46].

The study has been previously described in more detail elsewhere [46]. The Healthy Start study protocol was approved by the institutional review boards at the University of Rhode Island, Brown University, and the University of Connecticut. Baseline data from all 120 FCCHs and 374 children were used for this analysis. This information was collected from January 2016 to June 2018.

### 2.1. Measures

#### 2.1.1. FCCPs and Children Demographics

Trained study staff collected the FCCPs demographic information, through telephone surveys and during an in-person visit. The providers were classified as Latino or non-Latino according to their answer to the question on their ethnicity (“Do you consider yourself to be a Latino/Latina/Hispanic?”) [46]. The surveys also included questions assessing the FCCHs’ characteristics, such as hours of operation, number of children, average number of hours children spent daily at the FCCH [46], and the providers’ participation in the CACFP. Parents completed a demographic survey about their child’s information, including age, sex, ethnicity, and hours spent in childcare [46]. 

#### 2.1.2. Child Dietary Intake

Dietary intake of each child was collected according to the Dietary Observation in Child Care (DOCC), a reliable and valid observation technique for this population [47]. Each certified field staff member observed up to three children without interfering with the children’s daily routine. The field staff visually estimated the amount and type of foods and beverages children were served and ate, as well as any additional or remaining servings. Amount consumed was calculated using the following equation: amount consumed = (amount served ± amount added or wasted) − amount remaining. Information about recipes and mixed dishes was collected from the FCCPs [46]. All foods and beverages (except water) consumed by the children were recorded, and only foods offered by the FCCPs and consumed by the children were included in this analysis. Observations were scheduled with the FCCPs and conducted twice, either on consecutive or non-consecutive days. At baseline, field staff completed two days of dietary observations on 317 children in the FCCHs. The records were entered into the nutrition analysis software Nutrition Data System for Research (NDSR), software versions 2015–2018 [48].

#### 2.1.3. Food Groups and Food Items

**Figure 1:**
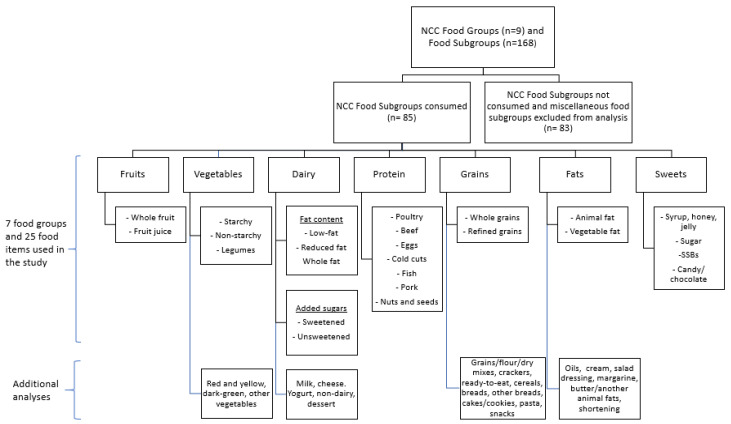
Aggregation process of the Nutrition Coordinating Center (NCC) food subgroups to determine food groups and food items.

The food groups and food items used for this analysis were based on the Nutrition Coordinating Center (NCC) food group classification and the NCC Food Group Serving Count System, which is the database that NDSR uses. This database classifies food into 168 subgroups, and each subgroup into one of 9 major food groups. A descriptive analysis was first run with the raw data. Of the 168 NCC food subgroups, 83 food subgroups were not consumed by children in FCCHs and were excluded from the analysis. Using the DGA 2015 as a reference [49], the NCC food subgroups consumed (*n* = 85) were aggregated to create the food subgroups used for this analysis, which will be referred henceforth on as the food items. This resulted in 25 food items distributed across 7 food groups (fruits, vegetables, dairy and non-dairy alternatives, proteins, grains, fats, and sweets). Figure 1 summarizes the aggregation process and the food groups and food items included. Fruits were grouped according to the way fruit was consumed (juice, whole fruit). Vegetables were grouped as starchy, non-starchy and legumes. Dairy and non-dairy alternatives were grouped according to fat and added sugar content; for example, low-fat and sweetened yogurt was classified as both low-fat dairy and sweetened dairy. For the protein group, NCC food subgroups representing similar foods/same type of meat were grouped; for example, poultry, lean poultry and fried chicken were aggregated to determine the contribution of poultry. The NCC food classification does not include sources of vegetable protein in the protein group. Grains were grouped as whole grain and refined; products with “some whole grains” products were considered refined. Fats were grouped as vegetable and animal fats. For sweets, the NCC food subgroups representing similar foods were grouped. Since foods were the primary aim of this study, beverages (e.g., soft drinks, fruit drinks, water) and the miscellaneous groups (e.g., pickled foods, gravy, sauces, condiments) were not included in this analysis. In order to analyze individual food items, additional subgrouping was conducted for vegetables, dairy and non-dairy alternatives, grains, and fats.

### 2.2. Statistical Analysis

#### 2.2.1. Food Item and Food Group Intake in FCCH 

Food intake was calculated as servings. Serving sizes (numbers of cups, ounces, and teaspoon equivalents) were defined by the NCC Food Serving Count System [48]. 

First, the children’s food item and food group intake were calculated at the FCCH level. For each item and group, all children’s intakes were averaged across the FCCHs to calculate a mean serving intake per FCCH. Mean serving intake was then averaged per FCCHs and by the FCCPs ethnicity [50].

#### 2.2.2. Contribution of Food Items to Major Food Groups 

The contribution of each food item to its respective major food group was calculated as a proportion, using ratio of means [50], and presented as a percentage. The mean ratio of each food item was calculated for each FCCH, and then averaged overall and by the FCCPs ethnicity. 

#### 2.2.3. Differences between Latino and non-Latino FCCPs

The differences in mean food group intakes and ratios between Latino and non-Latino FCCPs were tested with Analysis of Covariance (ANCOVA) models (*p* < 0.05). Bonferroni adjustments were made for multiple comparisons. Variables that were possible confounders and had a statistically significant association with the outcome (*p* < 0.1) were included in the adjusted model. The covariates considered for inclusion in the adjusted models were CACFP participation, FCCPs age, education and income, number of children in the home, the children’s age [12,19]. Children’s sex was not included since this was a home level analysis. SAS version 9.4 (SAS Institute, Inc., Cary, NC, USA) was used to carry out the analyses.

## 3. Results

### 3.1. Demographics

All FCCPs were female, with a mean age of 48.9 ± 9.0 years, and the majority (67.5%) identified themselves as Latino. Almost half of FCCPs (49.1%) reported an annual household income between USD 25,001–USD 50,000. On average, FCCPs cared for 7.7 ± 3.1 children in each FCCH and spent 62.4 ± 13.8 h per week working as FCCPs. Most providers (82.5%) participated in CACFP; however, only 7.5% participated in other programs such as Special Supplemental Nutrition Assistance Program (SNAP) and/or Women, Infants and Children (WIC). About half of the children were female (51.4%) and the majority were Latino (57.6%), with a mean age of 3.4 ± 0.9 years. On average, children were cared for 4.8 ± 0.8 days per week and spent 7.6 ± 0.9 h per day at the FCCHs (Table 1).

### 3.2. Food Group Intake

Overall, grains was the most consumed food group by children in FCCHs (0.63 ± 0.29), followed by fruit (0.37 ± 0.23), dairy (0.35 ± 0.18), protein (0.31 ± 0.25), fats (0.19 ± 0.17), vegetables (0.17 ± 0.14) and sweets (0.07 ± 0.18). Children cared for by Latino FCCPs had a significantly lower intake of total grain servings compared to children cared for by non-Latino FCCPs (0.60 ± 0.27 vs. 0.70 ± 0.32, *p* < 0.001). Significant differences by FCCPs ethnicity were not observed for any of the other food groups (Table 2).

### 3.3. Contribution of Food Items to Major Food Groups

Overall, the majority of fruit consumed was in the form of juice (85%). More than half of the vegetables consumed were non-starchy (61%), followed by legumes (24%), and starchy vegetables (15%) (Table 2). Dark-green vegetables represented only 7% of the vegetables consumed by children (Table 2). Most of the total dairy consumed was coming from low-fat dairy (70%). Most of the milk and yogurt consumed by the children was unsweetened (85%). In addition, it is worth noting that milk was the main contributor to dairy. For protein, poultry (44%) was the major contributor, with fish (5%) and pork (5%) being the least common food contributors. Three-quarters of the total grain group was refined (75%), with flour, dry mixes (28%), crackers (17%) and bread (15%) being the most common food items overall. Of the total fats consumed, vegetable fats (81%) contributed the most. Half of the sweets consumed in the FCCHs were syrup/honey/jelly (50%), followed by sugar (32%) and SSBs (12%). 

### 3.4. Differences between Latino and non-Latino FCCPs

There were differences by provider ethnicity in contributions of food items to food groups (Table 2). Children attending FCCHs with Latino FCCPs vs. non-Latino FCCPs ate a higher percent of legumes (33% vs. 5%, *p* < 0.001), poultry (48% vs. 34%, *p* < 0.001), grains, flour and dry mixes (34% vs. 13%, *p* < 0.001), and vegetable fats (oils) (89% vs. 63%, *p* < 0.001). Compared to FCCHs with non-Latino FCCPs, children cared for by Latino FCCPs had a lower proportion of fruit juice (82% vs. 90%, *p* < 0.001), non-starchy vegetables (52% vs. 82%, *p* < 0.001), red and yellow vegetables (23% vs. 29%, *p* < 0.001), other vegetables (23% vs. 45%, *p* < 0.001), low-fat dairy (68% vs. 77%, *p* < 0.001), nuts and seeds (5% vs. 21%, *p* < 0.001), snacks (2% vs. 8%, *p* < 0.001), butter and other animal fats (11% vs. 26%, *p* < 0.001), salad dressings (6% vs. 12%, *p* < 0.001), margarine (4% vs. 18%, *p* < 0.001) and cream (1% vs. 11%, *p* < 0.001).

## 4. Discussion

This study provides a more detailed insight into the food items that contributed to food group intake in preschool-aged children attending FCCHs. This study also found differences in the contribution of food items to children’s dietary intake between Latino and non-Latino FCCPs. These findings suggest FCCPs could increase vegetable variety, decrease fruit juice and whole grains offered to children in their FCCH. In addition, the differences in the food items found in this study suggest that the incorporation or reduction in specific food items can be reinforced during training or other educational sessions, and that Latino and non-Latino FCCPs could benefit from culturally tailored recommendations. Taken together, the results suggest that Latino FCCPs could be encouraged to provide more low-fat dairy, nuts and seeds. On the other hand, the non-Latino FCCPs, could increase whole fruits, legumes, nuts and seeds, vegetable fats, as well as reducing animal fats such as butter and cream intake. These findings support that the FCCPs ethnicity influences the foods offered in the FCCHs and highlights the importance of addressing cultural differences. 

The DGA and the CACFP recommend prioritizing whole fruit intake, however, this study shows that the major contributor to fruit consumed by children attending FCCHs was juice. This is in line with previous studies which have found a problematic, high contribution of juice in both childcare centers [35,40,51] and FCCHs [42,52], where fruit juice is served 3–4 times per week or more [52]. Furthermore, vegetables have been identified as a food group of concern in preschool age populations and childcare settings, due to insufficient variety and intake [35,39,40], low intakes of non-starchy vegetables and high intakes of starchy vegetables, especially fried potatoes [53]. However, in the current study, starchy vegetables were not the main source of vegetables, and instead, other vegetables and red/deep-yellow vegetables represented an important proportion. Dark-green vegetables contributed the least to the vegetable food group, as also shown in other studies [14,35,53]. This study found that children were consuming on average 0.63 servings of grains, from which three quarters was refined grains. CACFP guidelines encourage childcare settings to serve more than one serving/day of whole grains [54]; this is consistent with previous studies in childcare centers [14,35,55] and FCCHs [43], whereby children’s diets in childcare exceeded refined grain recommendations. 

Barriers to meeting CACFP and DGA guidelines are varied among food groups. Cost has been identified as a common barrier to purchasing healthy foods, specifically, whole fruits, vegetables and whole grains in childcare [44,56]. Child preferences and allergies have also been identified as a factor that affects the providers’ food choices [19], which may be related to the low intakes of fish and seafood and pork found in this study. Future studies and programs may consider focusing specifically on recommendations to increase nuts and seeds, fish and poultry. Along with this, CACFP guidelines state that unflavored low-fat (1% fat) or fat-free milk must be served [54], which is especially important when considering previous research pointing to high-fat milk as the major contributor of saturated fats in preschool-aged children [57,58]. While most of the dairy in this study was low-fat (1% fat), dairy with higher fat content was still being consumed. Offering low-fat milk is a feasible area for ongoing improvement in FCCHs considering that it is an important source of various nutrients [58]. 

This study did not analyze beverages as a major food group, however, SSBs were included in the sweet’s food group. SSBs have been identified as an important target to improve the nutrition environment within in childcare settings [59,60,61,62]; however, in this study, SSBs were not the main contributors to sweets. This is promising, given that SSB consumption in early childhood displaces more nutrient-dense foods and increases the likelihood of consuming them later in life [63]. In this study, the consumption of sweets was minimal, possibly due to most of the FCCPs participating in CACFP which recommends less added sugars. 

The current study found that the foods provided in FCCHs differed by providers’ ethnicity and by the composition of the foods included within each major food group. Even though there were no significant differences within the major food groups with the exception of grains, there were differences in the food items that comprised those major food groups: fruit juice, non-starchy vegetables, legumes, poultry, nuts and seeds, vegetable and animal fats. These findings highlight the importance of exploring the distribution or consumption of specific food items, support the influence of provider ethnicity in the foods offered in the childcare setting [22,25] and reinforce the need to assess the food environment, while addressing cultural differences [27]. 

Differences in practices between Latino and non-Latino FCCPs have been reported previously [22]. For example, Latino FCCPs have identified the importance of regulations, including CACFP, in influencing their nutrition related practices [17]. In addition, Latino FCCPs feel that it is their responsibility to provide children with nutritious foods [26]. Although previous studies have found that the broader Latino population in general consumes more fruit juice and animal fat relative to White populations [27,64,65], the finding that Latino FCCPs report being influenced by regulations and feeling responsible for serving nutritious foods may help explain the lower proportion of fruit juice and animal fat consumed in Latino FCCHs. Children who attended FCCHs with Latino FCCPs also consumed more legumes and less starchy vegetables as compared to FCCHs with Non-Latino FCCPs. This is consistent with previous studies whereby Latino populations consumed a greater intake of legumes when compared to other ethnicities [27,64]. Latino providers engage more in the preparation of home-cooked meals and mixed dishes [26] which may help explain the differences found in non-starchy vegetables. With regard to grains, children in FCCH with Latino FCCPs consumed more grains, flour and dry mixes, and less bread and snacks, as expected since rice is one of the main staple foods in the Latino community [66]. Tailored interventions to improve the food patterns and diet quality might benefit from understanding these differences, so that efforts can be focused on these specific food items. Results from this study might also reflect cultural preferences, for example, the lower proportion of nuts and seeds in the FCCHs with Latino FCCPs. 

Dairy was one exception in which Latino FCCPs were not following recommendations. Even though the CACFP guidelines state that low-fat dairy must be provided, a lower proportion of low-fat milk was served in the Latino FCCHs. Lower intakes of low-fat dairy among children overall have been described before, even after adjusting for CACFP participation [67]. It is possible that the selection of milk with higher fat content is driven by the perception that this is a healthier [68,69] and preferred option by children [70].

By focusing on individual food items, this study can help explain the differences in diet quality previously reported between Latino and non-Latino FCCHs. More specifically, significant differences were found for greens/beans, total protein, and seafood and plant protein, which can mainly be explained by the higher intake of legumes in the FCCHs with Latino providers. Findings from this study can also inform future research in FCCHs and can serve as guidance and contribute to the creation of creative and innovative programs. This is especially important when considering that education sessions should be actionable, simple, practical, by emphasizing suggestions for specific foods [17,53]. By identifying the differences in the food contributors between Latino and non-Latino FCCHs, more tailored recommendations and materials can be developed. 

Some limitations must be noted. The sample included FCCPs interested in participating in the study, from a specific area in the US, which may lead to selection bias and limit generalizability of the findings to some populations [71]. Further, scheduling the visits beforehand with the FCCPs could have influenced the food served during the observation day and introduce social desirability bias [72]. The NCC food classification and the aggregation process of the NCC subgroups to create food items used for this analysis could be slightly different from the one used in other studies; therefore, these differences should be addressed when comparing these data. Water intake was not collected as part of this study and may have been an important contributor to overall beverage intake beyond fruit juice. Future studies should consider collecting this data to assess overall beverage intake in FCCH. Lastly, it is important to consider that there might be additional cultural differences among both Latino and non-Latino FCCPs, such as country of origin. Future studies could explore differences within the Latino FCCPs to identify any other possible differences [71]. 

There were several strengths to this study. The more detailed insights into the types of foods consumed by the children in FCCHs settings can complement previous studies and help to better understand the nutrition environments in FCCHs, which is particularly important since most studies in childcare have been conducted in childcare centers. Differences between FCCHs and childcare centers, including economic and personnel resources, explain why in some cases, the interventions developed for childcare centers are not adaptable to FCCHs. Another strength was the use of the DOCC for the dietary data collection, which has been tested for validity and reliability in childcare settings [47]. Data were collected by trained personnel, therefore, did not rely on self-reported data, which has been a limitation in other studies [39,40,42], including those conducted on FCCHs [52,73,74]. Self-reported data can introduce self-report bias: it has been reported previously that when caregivers report what the children consumed, they tend to overestimate the quality of the diet and underreport foods considered unhealthy [75]. 

## 5. Conclusions

Foods items differed by FCCPs ethnicity, with children cared for by non-Latino FCCPs consuming a higher proportion of fruit juice, animal fats and a lower proportion of legumes. Children with Latino FCCPs consumed a lower proportion of non-starchy vegetables, low-fat dairy, and nuts/seeds. The findings from this study reinforce the need to identify strategies to improve foods consumed while at FCCHs and support previous studies that the provider’s ethnicity is related to the food items provided to the children. Latino and non-Latino FCCPs should decrease fruit juice, refined grains, include more fish and a higher variety of vegetables. According to the differences identified, non-Latino FCCPs should aim to increase the frequency of legumes and decrease animal fat. FCCHs with Latino FCCPs could benefit from the inclusion of more low-fat dairy, nuts and seeds, and more non-starchy vegetables. Cultural differences should be addressed to provide more tailored recommendations and trainings in food purchasing preparation.

## Figures and Tables

**Table 1:** Descriptive characteristics of the Family Childcare Home Providers (FCCPs) (*n* = 120) and the children (*n* = 374) enrolled in the Healthy Start/Comienzos Sanos study at baseline.

	Mean (SD) or *n* (%)	
Characteristics	Providers	Children
Sex, *n* (%)		
- Female	120 (100%)	180 (48.7%)
- Age, mean (SD)	48.9 (9.0)	3.43 (0.9)
- Ethnicity, *n* (%)*		
- Latino	81 (67.5%)	208 (57.6%)
- non-Latino	39 (32.5%)	153 (42.4%)
Marital status, *n* (%)		
- Single, never married	11 (9.2%)	
- Married or living with partner	90 (75.0%)	
- Divorced	10 (8.3%)	
- Separated/Widowed	9 (7.5%)	
Education level, *n* (%)		
- Less than high school diploma or GED	13 (10.9%)	
- High school diploma or GED	39 (32.5%)	
- More than high school	68 (56.6%)	
Annual Household Income, *n* (%)		
- Less than USD 25,000	16 (13.8%)	
- Between USD 25,001–USD 50,000	57 (49.2%)	
- Between USD 50,000–USD 75,000	24 (20.7%)	
- More than USD 75,001–USD 100,000	19 (16.3%)	
- Hours spent as an FCCP per week, mean (SD)	62.4 (±13.8)	
- Number of children enrolled per home, mean (SD)	7.7 (±3.1)	
CACFP Participation, *n* (%)		
- Yes	99 (82.5%)	
Program participation, *n* (%)		
- SNAP	4 (3.3%)	
- WIC	4 (3.3%)	
- WIC + SNAP	1 (0.9%)	
- None	111 (92.5%)	
Hours spent in childcare, mean (SD)		7.6 (0.9)
Days per week in the FCCH, mean (SD)		4.8 (0.8)

GED, General Education Diploma. SNAP, Supplemental Nutrition Assistance Program. WIC, Special Supplemental Nutrition Program for Women, Infants, and Children. CACFP, Child and Adult Care Food Program.

**Table 2:** Mean serving ^a^ intake of food items and food groups of children in Family Childcare Homes (FCCHs), the contribution (%) of food items to the major food groups and differences according to the ethnicity of the Family Childcare Providers (FCCPs).

	Overall (*n* = 120)		Latino Providers (*n* = 81)		Non-Latino Providers (*n* = 39)			
Food Group/ Food Items	Mean Serving (SD)	%	Mean Serving (SD)	%	Mean Serving (SD)	%	*p* Value for Means ^b^	*p* Value for % ^b^
FRUITS	0.37 (0.23)		0.40 (0.30)		0.35 (0.23)		0.31	
Whole fruit	0.29 (0.20)	15	0.10 (0.19)	18	0.04 (0.08)	10	0.26	0.05
Fruit juice	0.08 (0.16)	85	0.28 (0.20)	82	0.31 (0.23)	90	0.45	<0.001 *
VEGETABLES	0.17 (0.14)		0.18 (0.14)		0.14 (0.14)		0.17	
Non-starchy ^c^	0.09 (0.10)	61	0.08 (0.10)	52	0.11 (0.11)	82	0.01	<0.001 *
- Red and yellow	0.04 (0.06)	24	0.04 (0.05)	23	0.05 (0.08)	29	0.02	<0.001 *
- Dark green	0.01 (0.03)	7	0.01 (0.04)	7	0.01 (0.02)	7	0.19	0.85
- Other vegetables ^d^	0.04 (0.06)	29	0.03 (0.05)	23	0.05 (0.06)	45	0.02	<0.001 *
Starchy ^e^	0.03 (0.07)	15	0.04 (0.07)	15	0.03 (0.06)	13	0.31	0.16
Legumes	0.04 (0.06)	24	0.06 (0.07)	33	0.00 (0.00)	5	<0.001 *	<0.001 *
DAIRY/ ALTERNATIVE	0.35 (0.18)		0.33 (0.16)		0.38 (0.21)		0.01	0.01
Dairy products								
Milk	0.25 (0.04)	71	0.24 (0.13)	72	0.27 (0.21)	69	0.07	0.13
Cheese	0.05 (0.03)	14	0.05 (0.08)	13	0.07 (0.12)	16	0.52	0.36
Yogurt	0.04 (0.03)	11	0.04 (0.04)	12	0.02 (0.03)	9	0.72	0.65
Non-dairy	0.00 (0.01)	1	0.00 (0.02)	1	0.01 (0.03)	2	0.68	0.12
Desserts ^f^	0.01 (0.01)	2	0.00 (0.01)	1	0.01 (0.03)	4	0.82	0.62
According to fat content ^g^								
Low-fat	0.25 (0.17)	71	0.23 (0.15)	68	0.29 (0.21)	77	0.11	<0.001 *
Reduced	0.03 (0.07)	10	0.03 (0.06)	11	0.04 (0.08)	8	0.06	0.13
Whole fat	0.06 (0.08)	19	0.06 (0.09)	21	0.05 (0.06)	15	0.05	0.16
According to added sugar ^h^								
Sweetened	0.04 (0.05)	15	0.04 (0.04)	14	0.03 (0.05)	17	0.48	0.64
Unsweetened	0.25 (0.16)	85	0.24 (0.13)	86	0.27 (0.22)	83	0.24	0.53
PROTEIN	0.31 (0.25)		0.31 (0.24)		0.31 (0.26)		0.18	
Poultry	0.14 (0.14)	44	0.16 (0.20)	48	0.12 (0.16)	34	0.50	<0.001 *
Beef	0.05 (0.11)	14	0.05 (0.10)	15	0.06 (0.14)	12	0.76	0.84
Eggs	0.03 (0.06)	10	0.03 (0.06)	10	0.03 (0.05)	9	0.34	0.79
Cold cuts	0.04 (0.10)	12	0.03 (0.09)	10	0.05 (0.13)	16	0.02	0.22
Fish	0.01 (0.05)	5	0.02 (0.06)	6	0.01 (0.04)	4	0.60	0.87
Pork	0.01 (0.06)	5	0.01 (0.05)	5	0.02 (0.08)	4	0.31	0.35
Nuts and seeds	0.02 (0.06)	10	0.02 (0.05)	5	0.04 (0.08)	21	0.17	<0.001 *
GRAINS	0.63 (0.29)		0.60 (0.27)		0.70 (0.32)		<0.001 *	
Grain product								
Grain, flour, dry mixes ^i^	0.18 (0.07)	28	0.24 (0.18)	34	0.14 (0.29)	13	0.02	<0.001 *
Crackers	0.11 (0.05)	17	0.05 (0.07)	15	0.08 (0.11)	20	0.17	0.47
Ready-to-eat cereals	0.07 (0.04)	11	0.05 (0.06)	13	0.02 (0.04)	7	0.18	0.17
Bread	0.09 (0.04)	15	0.08 (0.10)	12	0.17 (0.15)	20	0.34	0.01
Other breads ^j^	0.08 (0.04)	12	0.09 (0.14)	9	0.17 (0.21)	16	0.60	0.05
Cakes/cookies ^k^	0.04 (0.03)	7	0.03 (0.07)	7	0.03 (0.07)	6	0.52	0.70
Pasta	0.04 (0.03)	7	0.05 (0.10)	6	0.06 (0.08)	8	0.71	0.61
Snacks ^l^	0.03 (0.03)	4	0.01 (0.03)	2	0.03 (0.05)	8	0.30	<0.001 *
Type of grain								
Whole grain	0.14 (0.16)	25	0.13 (0.15)	26	0.15 (0.18)	24	0.09	0.55
Refined grain	0.49 (0.29)	75	0.47 (0.28)	74	0.55 (0.30)	76	0.08	0.55
FATS	0.19 (0.17)		0.19 (0.15)		0.18 (0.21)		0.27	
Oils	0.05 (0.07)	59	0.12 (0.11)	73	0.05 (0.11)	27	<0.001 *	<0.001 *
Butter/other animal fats	0.03 (0.04)	16	0.03 (0.09)	11	0.04 (0.01)	26	0.50	<0.001 *
Salad dressings	0.01 (0.03)	7	0.00 (0.01)	6	0.01 (0.02)	12	0.16	<0.001 *
Margarine	0.02 (0.03)	8	0.02 (0.08)	4	0.05 (0.14)	18	0.10	<0.001 *
Shortening	0.01 (0.03)	5	0.01 (0.05)	5	0.01 (0.08)	6	0.17	0.15
Cream	0.01 (0.03)	5	0.00 (0.02)	1	0.01 (0.03)	11	0.39	<0.001 *
Vegetable vs. animal origin								
Vegetable	0.15 (0.15)	81	0.16 (0.13)	89	0.12 (0.19)	63	<0.001 *	<0.001 *
Animal	0.04 (0.08)	19	0.03 (0.09)	11	0.05 (0.06)	37	0.11	<0.001 *
SWEETS	0.07 (0.18)		0.07 (0.20)		0.06 (0.14)		0.07	
Syrup, honey, jelly	0.03 (0.03)	50	0.01 (0.03)	54	0.01 (0.02)	44	0.57	0.66
Sugar ^m^	0.02 (0.09)	32	0.02 (0.10)	30	0.02 (0.07)	37	0.09	0.09
SSBs	0.01 (0.03)	12	0.01 (0.03)	15	0.01 (0.03)	8	0.55	0.45
Chocolate/candy	0.00 (0.01)	5	0.00 (0.00)	2	0.00 (0.01)	6	0.10	0.03

SSBSs, sugar sweetened beverages. ^a^ A serving equals to: Fruits—medium unit; ½ cup fresh, frozen, cooked, canned fruit; ¼ cup dried fruit; 4 fluid ounces of juice. Vegetables—1 cup raw leafy vegetables; ½ cup other raw/cooked vegetables; 4 fluid ounces of juice. Grains—1 slice of bread; 1 ounce ready-to-eat cereal; ½ cup cooked cereal/rice/pasta. Protein—1-ounce cooked meat/fish/poultry; 1 egg; 1 tablespoon peanut butter; ½ ounce nuts/seeds. Dairy and non-dairy alternatives—1 cup milk/yogurt; 1 ½ ounces natural cheese; 2 ounces processed cheese. Fats—1 tsp margarine/oil/other animal fats; 30 g salad dressing; 30 g mayonnaise type dressing. Sweets—4 g sugar, 1 TB honey/jam; 15 g hard candies; 40 g all other candies. ^b^ *p* value for adjusted models. ANCOVA models compared differences between Latino and non-Latino providers. Covariates included in the adjusted models were CACFP participation, provider’s age, education and income, number of children in the home and children’s age. ^c^ Included dark green, deep yellow, tomato and other vegetables. ^d^ Includes raw, cooked and canned, stews, soups, pickles and pickled vegetables, relishes, salsas, mixed vegetables from other categories. ^e^ Included white potatoes, fried potatoes and other starchy vegetables. ^f^ Includes frozen dairy desserts, pudding and other dairy desserts and frozen non-dairy desserts. ^g^ Analysis included milk, yogurt, cheese. ^h^ Analysis included milk and yogurt. ^i^ Includes cooked grain/cereal, flour, cornmeal, bran or wheat germ, rice. ^j^ Includes quick breads, corn muffins, tortillas, French toast, waffles, pancakes, biscuits. ^k^ Includes cakes, cookies, pies, pastries, Danish, doughnuts and cobblers. ^l^ Includes snack chips, snack bars and popcorn. ^m^ Sugar refers to sugar entered in Nutrition Data System for Research (NDSR) as an ingredient of a homemade beverage or recipe. Does not include sugar from packaged products. * Significant after multiple comparison adjustment (*p* < 0.002).
